# Epidemiology of Anterior Cruciate Ligament Reconstruction Surgery in Italy: A 15-Year Nationwide Registry Study

**DOI:** 10.3390/jcm10020223

**Published:** 2021-01-10

**Authors:** Umile Giuseppe Longo, Kanto Nagai, Giuseppe Salvatore, Eleonora Cella, Vincenzo Candela, Francesca Cappelli, Massimo Ciccozzi, Vincenzo Denaro

**Affiliations:** 1 Department of Orthopaedic and Trauma Surgery, Campus Bio-Medico University, Via Alvaro del Portillo, 200, Trigoria, 00128 Rome, Italy; g.salvatore@unicampus.it (G.S.); v.candela@unicampus.it (V.C.); francescacappelli.8@gmail.com (F.C.); denaro@unicampus.it (V.D.); 2 Department of Orthopaedic Surgery, Kobe University Graduate School of Medicine, 7-5-1, Kusunoki-cho, Chuo-ku, Kobe, Hyogo 650-0017, Japan; kantona9@gmail.com; 3 Medical Statistics and Molecular Epidemiology, Campus Bio-Medico University, Via Alvaro del Portillo, 200, Trigoria, 00128 Rome, Italy; e.cella@unicampus.it (E.C.); m.ciccozzi@unicampus.it (M.C.)

**Keywords:** anterior cruciate ligament, epidemiology, registry, surgery, prevalence, reconstruction

## Abstract

There remains little information on the epidemiology of anterior cruciate ligament reconstruction (ACL-R), therefore, we performed an epidemiological evaluation on the ACL-R procedures performed in Italy from 2001 to 2015 to highlight potential disparities in access to healthcare. The National Hospital Discharge records (SDO) maintained at the Italian Ministry of Health were analyzed from 2001 to 2015; 248,234 ACL-Rs were performed in Italy over the 15-year study period in the adult population (starting from 15 years old), and the incidence rate per year in 100,000 persons ranged from 21.70 to 33.60 over the study period. The overall male/female ratio was 4.54. The length of hospitalization ranged from four days in 2001 to two days in 2015. Italy is historically divided into north, center, and south regions, and more than half of ACL-R surgery was performed in the north (67.2%); 95.2% of ACL-Rs were underwent in public institutions. The predicted model projected a slight growth in the number of ACL-Rs in the next 10 years (2016–2025). The number of ACL-R procedures increased in the adult population from 2001 to 2015. The ACL-R procedures were concentrated in the north of Italy, suggesting that efforts on regionalization of ACL-Rs should turn toward improving quality in hospitals in the south of Italy.

## 1. Introduction

The anterior cruciate ligament (ACL) is one of the most commonly injured ligaments of the knee [1], and ACL reconstruction (ACL-R) is widely accepted as the treatment of choice for individuals with functional instability due to ACL deficiency [2]. The literature on ACL-R has focused primarily on individual practice patterns, choice of graft type, surgical approach, and other technical aspects of the procedure [3,4]. Although the reports from large registry databases and a long-term cohort study have shown that the incidence of ACL-R procedures increased in the US [3,5,6] and other countries [5,6,7,8], there remains little information on the epidemiology of ACL-Rs. One of the founding principles of the Italian National Health Service (NHS) is fairness in access to healthcare. The Italian NHS is free to patients at the point of use. By evaluating national databases, which provide an overview of the epidemiological models of a disease, allowing through statistical inference to analyze the entire general population, we can better understand the epidemiological models of ACL-R procedures in Italy. In this way, we will be able to improve treatment strategies and health service planning in order to improve patients’ quality of life.

Therefore, the aim of the present study was (1) to perform an epidemiological evaluation on the ACL-R procedures performed in Italy from 2001 to 2015 and (2) to highlight potential disparities in access to healthcare in Italy and patients’ necessity to migrate among regions in order to obtain it by analyzing the national hospital discharge record (SDO) data of the Italian Ministry of Health.

## 2. Material and Methods

Data from national hospital discharge records (SDO) maintained at the Italian Ministry of Health for ACL repair (ICD-9-CM 81.45 or 81.43) from 2001 to 2015 were collected to perform this investigation (all data typologies contained in SDO’s related to ACL repair are shown in supplementary Table S1).

Since the Italian Ministerial Decree of 28 December 1991, SDO is the ordinary instrument for the collection of each patient’s information, derived from all Italian public and private hospitalization facilities. SDO contains anonymous data, including the patient’s age (evaluated in the ISTAT age group), sex, census region (region of residence), the region of hospitalization, length of hospitalization, public or private reimbursement, and diagnosis.

The Italian Ministry of Health has defined the mode of transmission of this information: each public or private hospital compiles the SDOs from the medical records of the patients discharged. Subsequently, on a monthly basis, each facility sends the data to the region. After carrying out the necessary checks, the region sends the data to the Ministry of Health within 45 days. 

The ICD-9-CM is an international system of classification of diagnoses and surgical and diagnostic-therapeutic procedures. It is used in the SDOs to code the corresponding information.

In this study, ACL surgery reconstruction was defined by the combination of the following ICD-9-CM codes:

- Diagnosis Code: 717.83 (old disruption of anterior cruciate ligament)
- Surgical Treatment Code: 81.45 (other repair of the cruciate ligaments) or 81.43 (triad knee repair).

The annual number of ACL repairs in the whole Italian population was calculated by analyzing the incidence rates, using the annual population size obtained from ISTAT (National Institute for Statistics). ISTAT divides the whole population into age classes starting at age 0 and ending at age 100+. Each class comprises five years (such as 0–4 years old, 5–9 years old, and so forth). So even though this study analyzes the adult population, data from those whose age was 15 years or older were included in the present study (starting from the age group 15–19 years old). The age group 0–14 years old was included in the pediatric and young adult population [9].

Therefore, considering this way of census and the frequency of data updating, the high information content allows the performance of important clinical-epidemiological analyses to evaluate the effectiveness of the assistance provided and potential health planning interventions [9,10,11,12,13].

### 2.1. Region of Hospitalization and Domicile of the Patients, Public or Private Surgery

**Figure 1:**
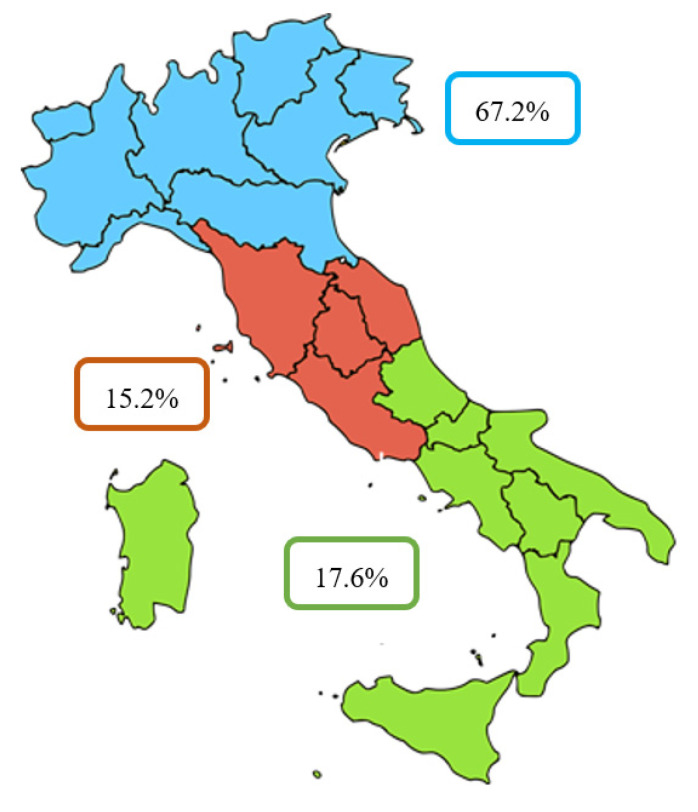
Percentage of ACL-Rs in Italian macro-regions.

A descriptive statistic to evaluate patients’ census region, the region of hospitalization, and region where surgery was performed. Procedures performed on patients residing in the same region of hospitalization were defined as regional surgeries. Procedures performed on patients not living in the same region of hospitalization were defined as extra-regional surgeries. The Italian regions were divided into three macro-regions: north, center, and south (Figure 1). 

The north includes the regions of the northwest (Liguria, Lombardy, Piedmont, and Aosta Valley) and those of the northeast (Emilia–Romagna, Friuli–Venezia Giulia, Trentino—South Tyrol, Veneto). The center includes the regions of Lazio, Marche, Tuscany, and Umbria. The south includes the regions of southern Italy (Abruzzo, Basilicata, Calabria, Campania, Molise, and Apulia) and the islands (Sardinia and Sicily).

Descriptive statistical analyses were used to calculate the annual number of ACL reconstruction in the whole Italian population. Incidence rates were calculated using the annual adult population size obtained from ISTAT. 

### 2.2. Projection

Projection of the trend in the number of ACL repairs in the next 10 years (2016–2025) was performed using the forecast function in Excel (Microsoft) software, including age and gender as variables. The data were treated as a time series, since they represent a set of data points that are measured at successive and uniformly spaced time intervals, in accordance with relevant guidelines and regulations. The forecasting function in the Excel software is based on linear regression. Linear regression is a linear approach to modelling the relationship between a dependent variable and one or more independent variables. Linear regression is the first type of regression analysis model that has been commonly used in the studies. It can easily fit the historical data and fit a predictive model to an observed data set better than other methods.

### 2.3. Cost Analysis

Analyses of ACL-R costs were based on the costs ascribed to diagnosis-related groups (DRGs) according to the ministerial decree (27 January 2010).

## 3. Results

### 3.1. Region of Hospitalization and Domicile of the Patients, Public or Private Surgery

There were 248,234 ACL-Rs performed in Italy during the study period. All of the epidemiological characteristics are described in Table 1.

**Figure 2:**
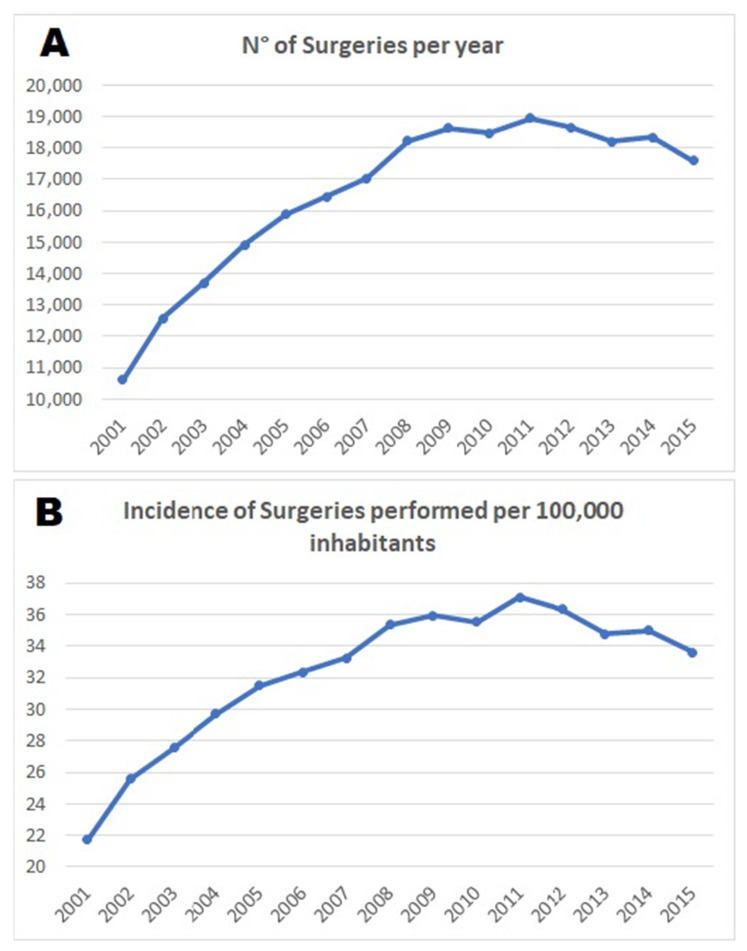
(**A**) Number of surgeries per year—the entire population; (**B**) incidence of surgeries performed per 100,000 inhabitants.

The total male/female ratio was 4.54, whereas the macro region of hospitalization was primarily the north of Italy with 67.2% of the surgeries. The median length of hospitalization ranged from 4.98 days in 2001 to 2.09 days in 2015. The cumulative period of incidence was 32.43 ACL-Rs for every 100,000 Italian adult inhabitants (Figure 2). 

The incidence rate per year out of 100,000 inhabitants ranged from 21.70 to 33.60 in the study period (2001–2015), with a peak of 37.11 in year 2011 and a light decrease in the last four years (2012–2015).

**Figure 3:**
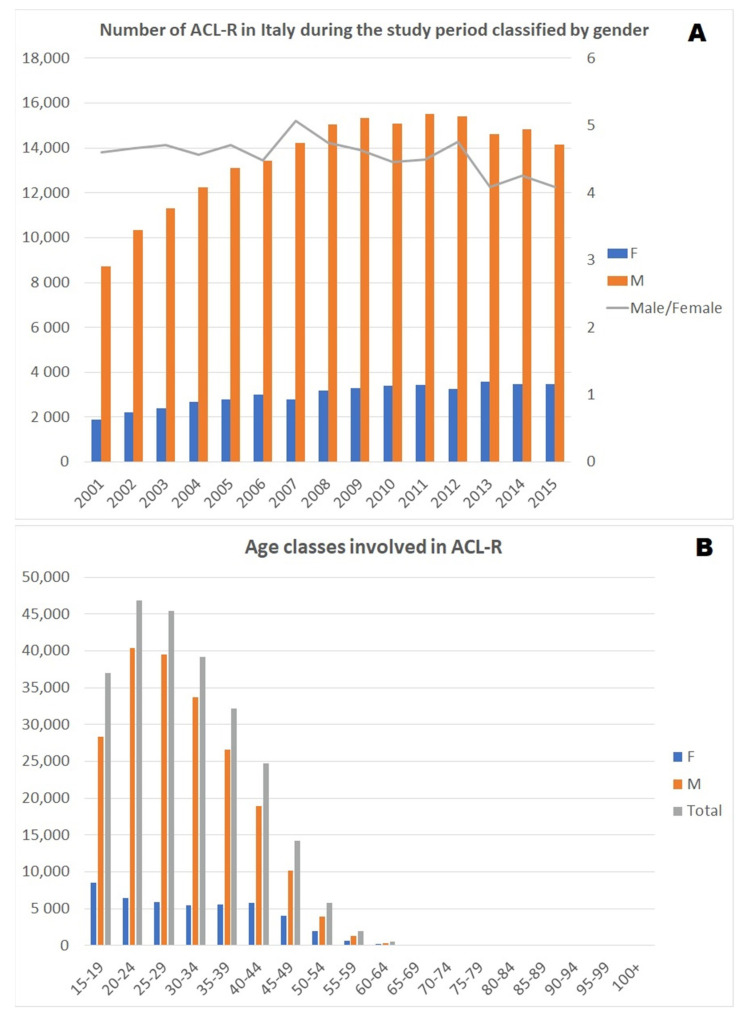
(**A**) Number of ACL-Rs in Italy during the study period classified by gender; (**B**) age classes involved in ACL-Rs.

The average male/female ratio was 4.54, implying that men were more subjected to this surgery. The male/female ratio increased each year, reaching a peak of 5.07 in 2007 and decreasing in the last few years (Figure 3A).

The distribution of the data looks like a curve skewed to the right; this means that there was a peak at the young age class (15–39 years) followed by a decrease in the elderly age classes (Figure 3B).

**Figure 4:**
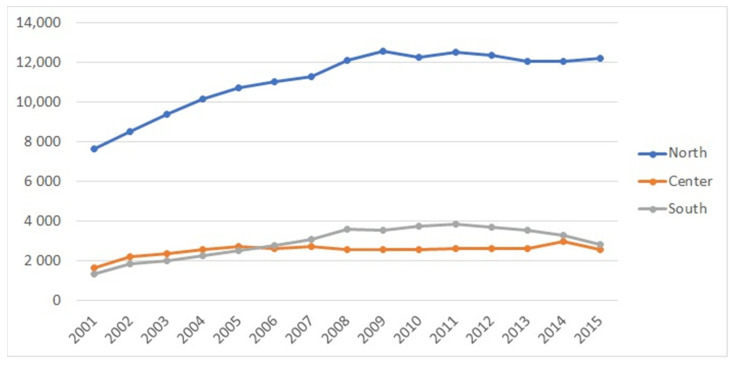
ACL-R surgery involvement regions.

The macro region most involved in the ACL-R surgery was the north (67.2%), followed by the south (17.6%) and center (15.2%) (Figure 4). 

In northern Italy, Lombardy, Emilia Romagna, and Veneto were found as the regions with the highest number of surgeries. It was not possible to determine the domicile region for 616 (0.25%) of the 248,234 surgeries.

**Figure 5:**
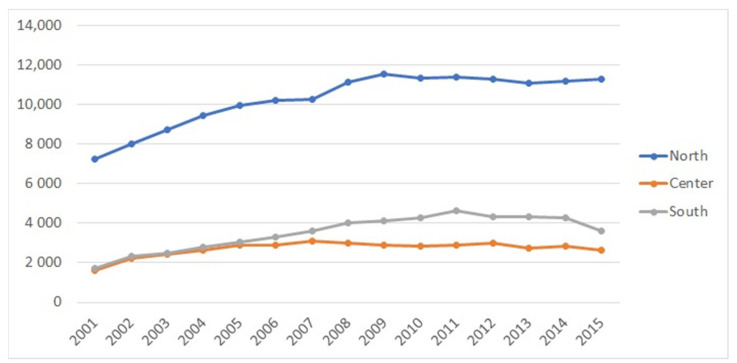
ACL-R surgery trend based on regional residence.

Patients living in the north mostly underwent surgeries in their region of residence (98.9%), whereas this tendency was less observed in the south (79.1%) and in the center of Italy (79.6%) (Table 2 and Figure 5).

Following the movement of the population, we have found that of 248,234 surgeries performed. In Table 3, it is evident that people living in the northern region of Italy preferred to have surgery in the same region, whereas the movement of the patient was mainly represented in the south toward the northern region of Italy, in the center of the country. The tendency was to have surgery in the same region or in the neighboring regions (see the diagonal in Table 3).

In the study period, the ratio of ACL-Rs performed in public institutions was 95.20%, and in the entire study period, this ratio was very similar to the average ratio of the whole period of observation.

### 3.2. Projection 

**Figure 6:**
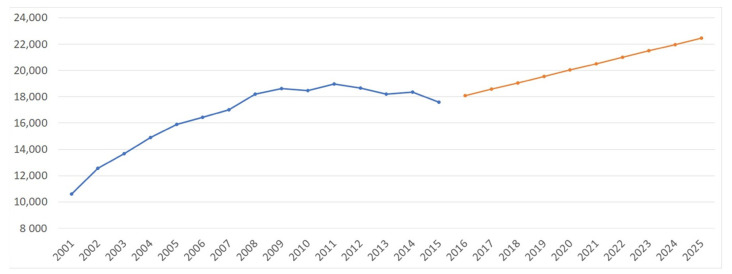
Projection analysis of ACL-Rs.

The projection model obtained with the Microsoft Excel forecast function analyzes the ACL-R data between 2001 and 2015 (shown in blue in Figure 6) to estimate future growth in the number of ACL-Rs over the next 10 years (2016–2025). In this regard, the expected growth is 24.16% in 10 years until this reaches a plateau around 2024–2025 (shown in orange in Figure 6). This means that there will be a 24.16% net increase in ACL surgeries in 2025 compared to 2016.

**Figure 7:**
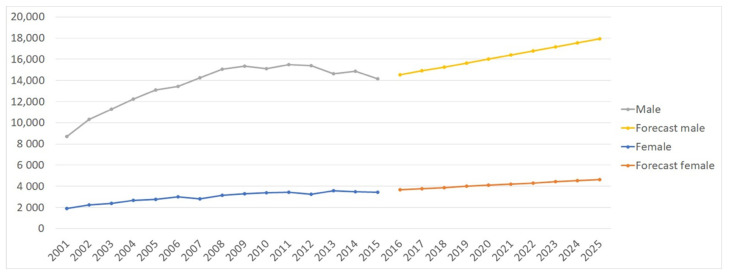
Projection analysis of ACL-Rs by gender.

**Figure 8:**
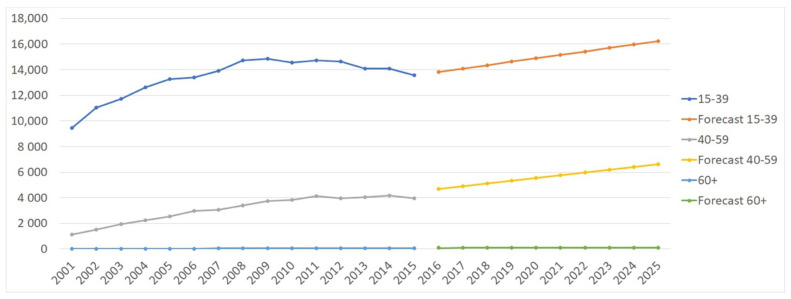
Projection analysis of ACL-Rs by age group.

The projection model shows that the number of ACL-Rs is expected to increase by 23.47% in males and 26.29% in females between 2016 and 2025 (Figure 7). However, despite the significant increase in the incidence of ACL-Rs in females, the prevalence of ACL-Rs remains consistently higher in males. Moreover, Figure 8 shows a predictable increase in ACL-Rs in all age groups, but particularly in the 40–59 and 60+ age groups.

### 3.3. Cost Analysis

The actual average hospital reimbursement was €1252.36 per ACL-R. Thus, about €310,878,332 has been spent for ACL-Rs in Italy between 2001 and 2015. In line with the forecast model, hospital costs borne by the NHS for ACL procedures are projected to be approximately €253,901,945 over the next 10 years (from 2016 to 2025).

For a more complete view to the Reader, the Results of this study are summarized in Table 4.

## 4. Discussion

The main finding of the present study is that the number of ACL-R procedures increased in the adult population between 2001 and 2015 in Italy, and the projection model predicted a slight increase in the number of surgeries until 2025. The incidence of ACL-Rs rose from 21.70 per 100,000 person-years in 2001 to 33.60 per 100,000 person-years in 2015, with a peak of 37.11 per 100,000 person-years in year 2011 and a light decrease in the last four years (2012–2015). The trend toward an increased number of ACL-R procedures was similar to previous studies in the US [3,5,6]. A study from New Zealand demonstrated an incidence of ACL-Rs of 36.9 per 100,000 person-years between 2000 and 2005 [5]. The cause of this upward trend in the frequency of ACL-Rs is likely multifactorial, and may represent an increasing activity level over time and a greater desire to return to an active lifestyle after an ACL injury. It may also represent an increase in the frequency of ACL injury, or in the likelihood of ACL-Rs should an injury occur, or both [3]. Another hypothesis could be that the new surgical techniques of ACL reconstruction allowed the treatment of patients who could not be treated in the traditional modality, such as patients with chronic unilateral ACL rupture and poor femoral bone quality [14,15,16]. Sanders et al. demonstrated that the incidence of new-onset ACL tears is decreasing over time among males, while remaining relatively stable in females from 1990 to 2010 [17].

In the whole adult population in Italy, the peak number of ACL-Rs was found in the 20–24 age group in both males and females in the present study. The results of the present investigations are in agreement with the literature, and confirm the higher prevalence of ACL-R interventions in men, although the incidence of ACL-Rs increased in females and decreased in men, still maintaining an overall male/female ratio of 4.54. In addition, the results of this study confirmed what has been described in the literature, emphasizing that ACL-R is performed primarily in the young population [18]. Moreover, a wide range of incidence rates of ACL-Rs has been published [3,5,6,17,18]; the incidence rates of ACL injury and ACL-Rs depend on the population being studied, and are likely higher in athletic populations compared to the population as a whole [18,19].

Interestingly, over this 15-year study period, the geographical variations in ACL-R procedures were present among Italian macro-regions; 67.2% of ACL-R surgeries were performed in the north, 15.2% in the center, and 17.6% in the south, which is a similar trend to the incidence of surgeries for rotator cuff tears in Italy [13]. Furthermore, those who lived in the northern region of Italy preferred to have surgery in the same region, whereas the movement of the patient is mainly represented in the south toward the north of Italy. In the view of the government and the Italian Ministry of Health’s focus on reducing health inequalities, the difference in ACL-R surgeries among regions of Italy reflects the heterogeneity of the care provided in different regions in Italy. It is known that the need is greater among the most deprived groups, and that education and income are unrelated to willingness to undergo surgery [20]. It is also well-known that, although a trend toward more frequent operative management of ACL damage was demonstrated [21], a variation in the management of these injuries persists [22]. It is probably for this last reason that citizens from less efficient regions are willing to travel from their home (and regional healthcare system [23]. Furthermore, these results seem to point out that getting patients from other regions may improve the quality of care for residents. In some ways, this is an interesting result, since it suggests that better quality of care is achieved because of patients’ mobility rather than competition in the internal market [23].

The length of hospitalization decreased from four days in 2001 to two days in 2015 in Italy, which is a similar finding to the length of hospital stay for the surgery of rotator cuff tears in Italy. This is somewhat expected, given the fact that hospitals have tried to shorten the length of hospitalization as much as possible under intense financial pressure.

The total amount of ACL-R procedures in private hospitals remained consistent over the study period, and most of the surgeries were performed in public hospitals. The main reason is that one of the founding principles of the Italian NHS is fairness in access to healthcare, and it is free for the patients at the point of use. The strength of the present study included the nationwide coverage, long time period, use of reliable nationwide data, and that the study population comprised the entire adult population of Italy, since data collection for the Italian Ministry of Health is mandatory for all hospitals, including public and private hospitals.

The Italian healthcare system follows the Beveridge model, where private healthcare activity complements but does not replace public healthcare guaranteed by the state. The cost analysis shows that surgical ACL repair is an average cost procedure, especially when there are no post-surgical complications and when no pathologies are associated. To compare with the United States, in which the national healthcare model is private, here the total cost of an ACL repair averages $2039.09 [24]. Considering that between approximately 100,000 and 130,000 ACL surgeries are performed each year in the United States [18] and that the cost per single ACL-R in the United States is higher than in Italy, the same money is spent each year in the United States as was used in 15 years (2001–2015) in Italy for ACL-Rs.

Several limitations should be noted. First, potential errors in data entry may exist due to the nature of database studies and does not relate to authors’ errors in data collection. However, this can exist in any national database. Second, there is a possibility that changes in coding practices could have impacted the current results. That means we are unaware of any systematic changes in coding of ACL-Rs, which would have significantly impacted the current findings. As with any health outcome study based on administrative data, our study had limitations with regard to the very nature of the data. We lacked clinical information, such as the mechanism of injury, operative details, complexity of the procedure, and rehabilitation protocol.

## 5. Conclusions

The number of ACL-R procedures increased in the adult population from 2001 to 2015, and the projection model predicted a slight increase in the number of surgeries until 2025 in Italy. The ACL-R procedures were concentrated in the northern region of Italy. The findings suggest that efforts on regionalization of ACL-Rs should turn towards improving quality in hospitals in the south of Italy, and there is evidence of inequity in access to ACL-Rs across the macro regions of Italy. 

## Figures and Tables

**Table 1:** Data on demographics.

	**2001**	**2002**	**2003**	**2004**	**2005**	**2006**	**2007**	**2008**	**2009**	**2010**	**2011**	**2012**	**2013**	**2014**	**2015**	**TOTAL**	
Total population ≥ 15 year old (ISTAT database)	48,884,353	49,172,932	49,697,896	50,206,663	50,467,775	50,809,387	51,252,247	51,513,562	51,862,391	52,003,159	51,068,990	51,336,889	52,334,535	52,412,490	52,383,692	765,406,961	
Number of hospitalizations	10,607	12,568	13,696	14,917	15,884	16,438	17,033	18,220	18,637	18,482	18,954	18,658	18,200	18,337	17,603	248,234	
Male	8716	10,348	11,296	12,235	13,105	13,442	14,227	15,048	15,331	15,098	15,507	15,416	14,627	14,852	14,142	203,390	
Female	1891	2220	2400	2682	2779	2996	2806	3172	3306	3384	3447	3242	3573	3485	3461	44,844	
Male/female ratio	4.61	4.66	4.71	4.56	4.72	4.49	5.07	4.74	4.64	4.46	4.50	4.76	4.09	4.26	4.09	4.54	
Region of hospitalization																	
North	7622	8518	9387	10,160	10,702	11,032	11,269	12,107	12,558	12,233	12,506	12,348	12,063	12,065	12,207	166,777	67.2%
Center	1647	2197	2338	2531	2690	2623	2701	2549	2535	2533	2594	2619	2629	2977	2565	37,728	15.2%
South	1338	1853	1971	2226	2492	2783	3063	3564	3544	3716	3854	3691	3508	3295	2831	43,729	17.6%
Domicile of hospitalized patients																	
From the north	7258	7991	8745	9427	9940	10,190	10,268	11,132	11,554	11,341	11,380	11,294	11,063	11,158	11,300	154,041	62.2%
From the center	1599	2229	2415	2655	2890	2885	3087	3013	2914	2849	2915	2977	2764	2850	2619	40,661	16.4%
From the south	1737	2321	2508	2800	3023	3325	3620	4026	4124	4255	4612	4336	4320	4277	3632	52,916	21.4%
	**2001**	**2002**	**2003**	**2004**	**2005**	**2006**	**2007**	**2008**	**2009**	**2010**	**2011**	**2012**	**2013**	**2014**	**2015**	**TOTAL**	
Incidence of surgeries performed per 100,000 inhabitants	21.70	25.56	27.56	29.71	31.47	32.35	33.23	35.37	35.94	35.54	37.11	36.34	34.78	34.99	33.60	32.43	
Public hospitalizations per 100,000 inhabitants	21.14	24.45	26.46	28.17	29.77	30.68	31.66	33.62	34.04	33.78	35.21	34.58	32.87	33.42	32.16	30.88	
Private hospitalizations per 100,000 inhabitants	0.56	1.11	1.09	1.54	1.70	1.67	1.57	1.75	1.88	1.74	1.91	1.76	1.91	1.57	1.44	1.55	
	**2001**	**2002**	**2003**	**2004**	**2005**	**2006**	**2007**	**2008**	**2009**	**2010**	**2011**	**2012**	**2013**	**2014**	**2015**	**TOTAL**	
Hospitalization length (days)	4.98	4.35	3.97	3.67	3.42	3.28	3.08	2.92	2.81	2.70	2.58	2.43	2.31	2.15	2.09	3.00	

**Table 2:** Migratory flows between regions.

Macro-Region of Residence	Macro-Region of Hospitalization	Frequency	Percent
Missing data	North	442	71.8
	Center	108	17.5
	South	66	10.7
	Total	616	100.0
North	North	152,350	98.9
	Center	1306	0.8
	South	385	0.2
	Total	154,041	100.0
Center	North	6868	16.9
	Center	32,366	79.6
	South	1427	3.5
	Total	40,661	100.0
South	North	7117	13.4
	Center	3948	7.5
	South	41,851	79.1
	Total	52,916	100.0

**Table 3:** Data on regions.

		Patient’s Origin																					TOTAL for Each Origin
2001–2015 TIME SPAN		Abruzzo	Basilicata	Calabria	Campania	Emilia Romagna	Friuli Venezia Giulia	Lazio	Liguria	Lombardy	Marche	Molise	A.P. of Bolzano	A.P. of Trento	Piedmont	Apulia	Sardinia	Sicily	Tuscany	Umbria	Aosta Valley	Veneto	
**Region of Hospitalization**	**Abruzzo**	2024	3	8	75	6	1	992	1	8	102	118	1	0	5	82	3	7	9	2	0	8	3462
	**Basilicata**	0	281	8	43	0	0	1	0	1	0	1	0	0	0	50	0	0	1	0	0	0	386
	**Calabria**	0	1	1141	4	3	2	7	1	3	1	0	0	0	3	5	1	14	4	0	1	0	1191
	**Campania**	19	94	150	11,781	29	16	169	7	50	7	25	4	5	16	330	8	19	36	2	2	11	12,802
	**Emilia Romagna**	249	128	546	436	19,943	154	259	122	989	3079	82	25	67	153	904	181	836	1348	369	6	1816	31,836
	**Friuli Venezia Giulia**	2	3	4	4	8	3773	9	0	15	1	0	2	2	4	5	0	36	4	3	0	1810	5693
	**Lazio**	181	103	287	662	45	13	14,500	36	76	165	70	0	11	40	500	51	246	302	155	6	42	17,565
	**Liguria**	3	0	12	8	9	0	7	2329	7	0	0	1	1	57	16	6	7	385	1	7	3	2867
	**Lombardy**	136	55	319	534	1717	95	150	1206	53,732	460	14	76	180	2704	413	95	783	438	33	131	734	64,155
	**Marche**	345	11	13	33	604	0	90	1	8	3131	16	1	0	6	80	1	20	5	29	0	7	4406
	**Molise**	36	2	3	98	7	0	19	0	5	2	884	0	0	2	435	0	0	1	1	0	1	1496
	**A.P. of Bolzano**	0	0	5	3	8	16	14	4	13	1	0	3184	290	4	14	5	7	29	1	0	117	3725
	**A.P. of Trento**	1	0	2	4	4	2	6	3	31	1	0	36	1862	2	4	1	6	5	2	0	194	2174
	**Piedmont**	5	7	124	22	38	7	18	607	1278	8	0	0	3	18,859	53	28	85	41	2	401	18	21,656
	**Apulia**	8	534	162	40	20	14	30	1	31	6	2	2	0	13	10,174	2	10	10	0	0	14	11,092
	**Sardinia**	1	1	0	1	6	5	4	2	17	2	0	4	0	6	1	5518	4	3	0	0	4	5582
	**Sicily**	1	2	238	8	4	2	8	3	15	4	0	1	2	11	3	1	7387	4	0	1	8	7718
	**Tuscany**	36	91	149	336	55	3	929	181	94	63	11	1	6	24	101	32	98	10,499	240	0	21	12,997
	**Umbria**	93	10	12	18	8	1	327	1	7	295	28	2	1	3	297	0	17	142	1494	0	2	2760
	**Aosta Valley**	0	1	0	1	2	1	2	3	8	0	0	1	0	209	0	2	3	3	0	613	0	851
	**Veneto**	45	35	58	224	884	2271	90	16	723	26	26	214	1353	27	337	34	158	64	9	3	27,163	33,820
**TOTAL for each Region**		3185	1362	3241	14,335	23,400	6376	17,631	4524	57,111	7354	1277	3555	3783	22,148	13,804	5969	9743	13,333	2343	1171	31,973	

**Table 4:** Summary of results.

Number of Hospitalizations	248,234
Male/Female Ratio	4.54
Region of Hospitalization (%)	North: 67.2% Center: 15.2% South: 17.6%
Incidence of Surgeries performed per 100,000 inhabitants	2001: 21.7 2015: 33.60 Total: 32.43
Hospitalization length (days)	2001: 4.98 2015: 2.09 Total: 3.00
Surgeries in the region of residence	North: 98.9% Center: 79.6% South: 79.1%
Burden	Private: 4.8% Public: 95.2%
Expected growth of ACL-Rs from 2016 to 2025	24.16%
Hospital costs between 2001 and 2015	310,878,332 euros
Hospital costs expected from 2016 to 2025	253,901,945 euros

## Data Availability

The data presented in this study are available on request from the corresponding author from Direzione Generale della Programmazione Sanitaria–Banca Dati SDO. The availability of these data is restricted and is therefore not available to the public.

## Supplementary Materials

The following are available online at https://www.mdpi.com/2077-0383/10/2/223/s1, Table S1: Kind of data contained in the SDO registries of the Italian Ministry of Health regarding ACL injury and treatment.
