# Supplementary material for: Epidemiology of Anterior Cruciate Ligament Reconstruction Surgery in Italy: A 15-Year Nationwide Registry Study
Source: J Clin Med. 2021 Jan 10;10(2):223. doi: 10.3390/jcm10020223 (PMC7826510; doi:10.3390/jcm10020223)
Supplement: Supplementary file 1 [file jcm-10-00223-s001.pdf]

Table S1. Kind of data contained in the SDO registries of the Italian Ministry of Health regarding ACL injury and treatment.

**Years**		**From 2001 to 2015**
Age		≥ 15 years
Sex		Male, Female
Region of hospitalization		Liguria, Lombardy, Piedmont, Aosta Valley, Emilia–Romagna, Friuli–Venezia Giulia, Trentino—South Tyrol, Veneto, Abruzzo, Basilicata, Calabria, Campania, Molise, Apulia, Sardinia and Sicily
Macro-region of hospitalization		North, Center and South
Region of resident		Liguria, Lombardy, Piedmont, Aosta Valley, Emilia–Romagna, Friuli–Venezia Giulia, Trentino—South Tyrol, Veneto, Abruzzo, Basilicata, Calabria, Campania, Molise, Apulia, Sardinia and Sicily
Macro-region of resident		North, Center and South
Time of hospitalization		Days of hospitalization
Burden		Private, Public
Primary diagnosis code		717.83
Main other diagnosis codes		717.2<br>717.3<br>717.40<br>717.43<br>717.49
Primary procedure codes		81.43<br>81.45
Main other procedure codes		80.6<br>78.45<br>80.26<br>83.41<br>89.52
Diagnosis	717.83	Old disruption of anterior cruciate ligament
	717.2	Derangement of posterior horn of medial meniscus
	717.3	Other and unspecified derangement of medial meniscus
	717.40	Derangement of lateral meniscus, unspecified
	717.43	Derangement of posterior horn of lateral meniscus
	717.49	Other derangement of lateral meniscus
Procedure	81.43	Triad Knee Repair
	81.45	Other Repair of the cruciate ligaments
	80.6	Excision of semilunar cartilage of knee
	78.45	Other repair or plastic operations on bone, femur
	80.26	Arthroscopy, knee
	83.41	Excision of tendon for graft
	89.52	Electrocardiogram
